# Interleukin 27 Signaling in Rheumatoid Arthritis Patients: Good or Evil?

**DOI:** 10.3389/fimmu.2021.787252

**Published:** 2022-01-04

**Authors:** Liang Han, Zhe Chen, Kun Yu, Jiahui Yan, Tingting Li, Xin Ba, Weiji Lin, Yao Huang, Pan Shen, Ying Huang, Kai Qin, Yinhong Geng, Yafei Liu, Yu Wang, Shenghao Tu

**Affiliations:** ^1^ Department of Integrated Traditional Chinese and Western Medicine, Tongji Hospital of Tongji Medical College of Huazhong University of Science and Technology, Wuhan, China; ^2^ Department of Cardiology, Tongji Hospital of Tongji Medical College of Huazhong University of Science and Technology, Wuhan, China; ^3^ Department of Nephrology, The First Affiliated Hospital of Zhengzhou University, Zhengzhou, China

**Keywords:** interleukin 27 (IL-27), rheumatoid arthritis, CD4^+^ T cells, synovial fibroblasts (FLS), osteoclasts (OCs), ectopic-like structure (ELS), rheumatoid nodules

## Abstract

The occurrence and development of rheumatoid arthritis (RA) is regulated by numerous cytokines. Interleukin 27 (IL-27) is a soluble cytokine that exerts biological effects by regulating the Janus tyrosine kinase (JAK)/signal transducer and activator of the transcription (STAT) signaling pathway *via* the IL-27 receptor. IL-27 is known for its pleiotropic roles in modulating inflammatory responses. Previous studies found that IL-27 levels are elevated in RA blood, synovial fluid, and rheumatoid nodules. Cellular and animal experiments indicated that IL-27 exerts multiple regulatory functions in RA patients *via* different mechanisms. IL-27 inhibits ectopic-like structure (ELS) formation and CD4^+^ T helper type 2 (Th2) cell, CD4^+^ T helper type 17 (Th17) cell, and osteoclast differentiation in RA, contributing to alleviating RA. However, IL-27 promotes Th1 cell differentiation, which may exacerbate RA synovitis. Moreover, IL-27 also acts on RA synovial fibroblasts (RA-FLSs) and regulatory T cells (Tregs), but some of its functions are unclear. There is currently insufficient evidence to determine whether IL-27 promotes or relieves RA. Targeting IL-27 signaling in RA treatment should be deliberate based on current knowledge.

## Introduction

Rheumatoid arthritis (RA) is a systemic immune disease characterized by chronic symmetrical synovitis inflammatory polyarthritis of the small joints (1). In addition to arthritis, RA causes various extra-articular complications, such as RA-associated interstitial lung disease (RA-ILD) and skin damage (2, 3). Specific environmental and genetic factors cause immune system disorders in RA patients and determine the occurrence and development of RA (4).

Various immune cells and multiple cytokines produced by immune cells exert essential roles in the development and disease progression of RA. Interleukins (ILs) are a class of cytokines widely involved in immune system modulation and are implicated in RA. Two CD4^+^ T cell subsets, T helper 1 cells (Th1) and T helper 17 cells (Th17), have long been considered risk factors for RA development (5). In contrast, T helper 2 cells (Th2) and regulatory T cells (Tregs) are decreased in number and exhibit functional impairment in RA. IL-1 and IL-17, which are secreted by Th1 and Th17 cells, respectively, have been reported to be proinflammatory factors in RA synovial membranes. In addition, IL-15, secreted by macrophages, promotes T cells in RA synovial membrane tumor necrosis factor (TNF)-α production in a cell contact-dependent manner (6). The cytokine network, constituted by multiple cytokines and feedback loops, subtlety regulates the immune response and inflammation in complex ways and is involved in RA development (7).

IL-27 is a pleiotropic immunoregulatory cytokine that was first identified in 2002 (8). Since the discovery of IL-27, its immune modulatory function has been gradually elucidated by numerous studies. An association between IL-27 single-nucleotide polymorphisms (SNPs) and genetic susceptibility to RA has been described (9). Abnormal IL-27 levels have been well demonstrated in previous studies, and IL-27 participates in RA development *via* multiple pathways (10–12). Earlier studies found that IL-27 primarily affects the differentiation of helper T cell subsets (13). However, more recent studies suggest that IL-27 might contribute to the pathogenesis of RA through additional direct and indirect regulatory pathways. Current studies have shown that IL-27 is extensively and profoundly involved in immunological imbalance in RA. Furthermore, IL-27 is a potential therapeutic target for the treatment of immune-related diseases. This review discusses updates on the regulatory effects of IL-27 on immune cells and RA development. We expect that our review may provide ideas for future studies, which would promote increased awareness of the relationship between RA and IL-27.

## The Components and Expression of IL-27 and the IL-27 Receptor (IL-27R)

**Figure 1:**
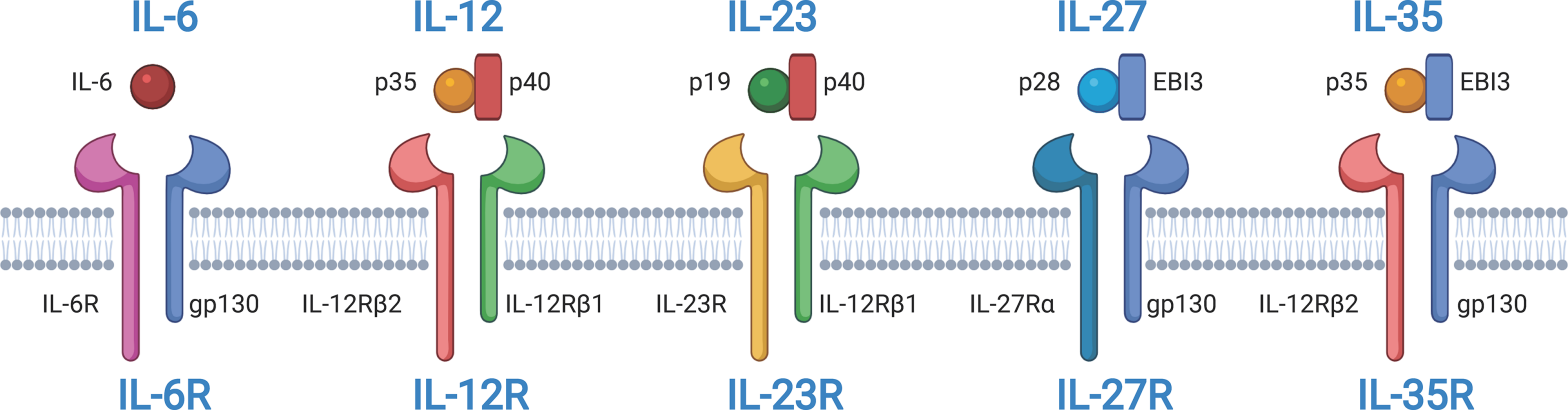
Structures of interleukin-12 (IL-12) and IL-6 cytokine families and their receptors. IL-6, IL-12, IL-23, IL-27, and IL-35 belong to IL-12 and IL-6 cytokine families. IL-12, IL-27, and IL-35 are composed of two subunits. IL-12 and IL23 share the common subunit p40. IL-12 and IL-35 share the common subunit p35. IL-27 and IL-35 share the common subunit Epstein-Barr virus induced 3 (EBI3). All receptors of IL-6, IL-12, IL-23, IL-27, and IL-35 are heterodimers. Some subunits are shared among receptors. IL-6R, IL-27R, and IL-35R share the common receptor subunit gp130. IL-12 and IL-23 share the common receptor subunit IL-12Rβ1. IL-12R and IL-35R share the common receptor subunit IL-12Rβ2. The figure was created with BioRender.com.

The IL-27 receptor (IL-27) is a member of the IL-12 and IL-6 cytokine families and was identified two decades ago  (8, 14, 15). IL-27 is a heterodimer with p28, and Epstein–Barr virus induced 3 (EBI3) subunits that are encoded by *IL27* and *EBI3*, respectively (**Figure 1**). The IL-27 p28 subunit is homologous to IL-12 p35 and IL-6  (16). EBI3 was first identified in B lymphocytes transfected with Epstein–Barr virus (EBV), and it is homologous to IL-12 p40  (17). However, the two subunits can exist independently. The IL-27 p28 subunit, also known as IL-30, can be secreted alone  (18). In this review, we use the designation “IL-27 p28 subunit” instead of “IL-30” because the name of the gene encoding the IL-27 p28 subunit protein is IL-27 according to HGNC guidelines  (8).

Existing studies suggest that recombinant IL-27 p28 blocks the activity of cytokines, including IL-6, IL-11, and IL-27  (16), which suppresses hepatic injury and alleviates experimental autoimmune uveitis  (19). However, other studies found that the conformation of IL-27 p28 used in some studies was incorrect. IL-27 p28 can bind with soluble IL-6Rα and signal in the presence of a more appropriate conformation. IL-27 p28 can even signal independently at high concentrations  (20, 21). This feature of IL-27 p28 raises a concern about IL-27, as we do not know whether IL-27 or IL-27 p28 is performing these biological functions. Further investigation is warranted to determine whether another subunit of IL-27, EBI3, can exert some of its biological functions independently. Determining the answers to these questions could help to better understand the roles of IL-27 in diseases.

IL-27R, a heterodimer, is a member of the class I cytokine receptor family, which is composed of α subunits of the cytokine binding protein and β subunits of the signal transduction protein (**Figure 1**) (14, 15, 22). The two subunits, IL-27Rα, also known as T cell cytokine receptor (TCCR) or WSX-1, and glycoprotein 130 (gp130) are composed of IL-27R, and they are both members of the immunoglobulin superfamily. Gp130 is a receptor subunit that is shared by IL-6 family cytokines. Both IL-27Rα and gp130 are necessary for IL-27 signal transduction. Memory CD8^+^ T cells are not responsive to IL-27 stimulation because they downregulate the expression of gp130  (23). Moreover, research has indicated that IL-27Rα is capable of forming homodimers and activates Janus tyrosine kinase (JAK)/signal transduction and activator of transcription (STAT) signaling  (24, 25). Additionally, soluble IL-27Rα is able to inhibit IL-27 signaling  (26). Similar to IL-27, these features of IL-27R also influence our understanding of IL-27 signaling in diseases.

IL-27Rα is expressed by several varieties of immune cells and non-immune cells, including T and B lymphocytes, NK cells, monocytes, macrophages, and hepatocytes  (27). It has been reported that lymphoid tissues, including the spleen, thymus, and lymph nodes, also express high levels of IL-27Rα  (28). Most cells express gp130, and its expression levels are related to cellular status  (29). Cells in different states may express different levels of IL-27. Cell activation causes CD4^+^ T cells to express increased IL-27Rα but causes NK cells to express reduced levels, which influences cellular responses to IL-27 signaling.

## Sources and Signaling Pathways of IL-27

IL-27 is a soluble-secreted cytokine, so understanding its cellular sources is important. The two subunits of IL-27 are expressed by many immune cells, such as monocytes/macrophages, lymphocytes, activated dendritic cells (DCs), plasma cells, natural killer (NK) cells, and other non-immune cells, such as placental trophoblasts and endothelial cells  (30, 31). Specific signaling stimulation also regulates IL-27 expression. Dibra et al. found that coexisting Toll-like receptor (TLR) 9 signaling from macrophages and CD3 signaling from T cells stimulate the expression of IL-27 p28 in splenic cells  (32). Liu et al. determined that lipopolysaccharide (LPS) and interferon-γ stimulation promotes IL-27 p28 expression in macrophages  (30). Moreover, in DCs, expression of EBI3 was induced by TLR signaling *via* nuclear factor kappa B (NF-κB)  (33).

**Figure 2:**
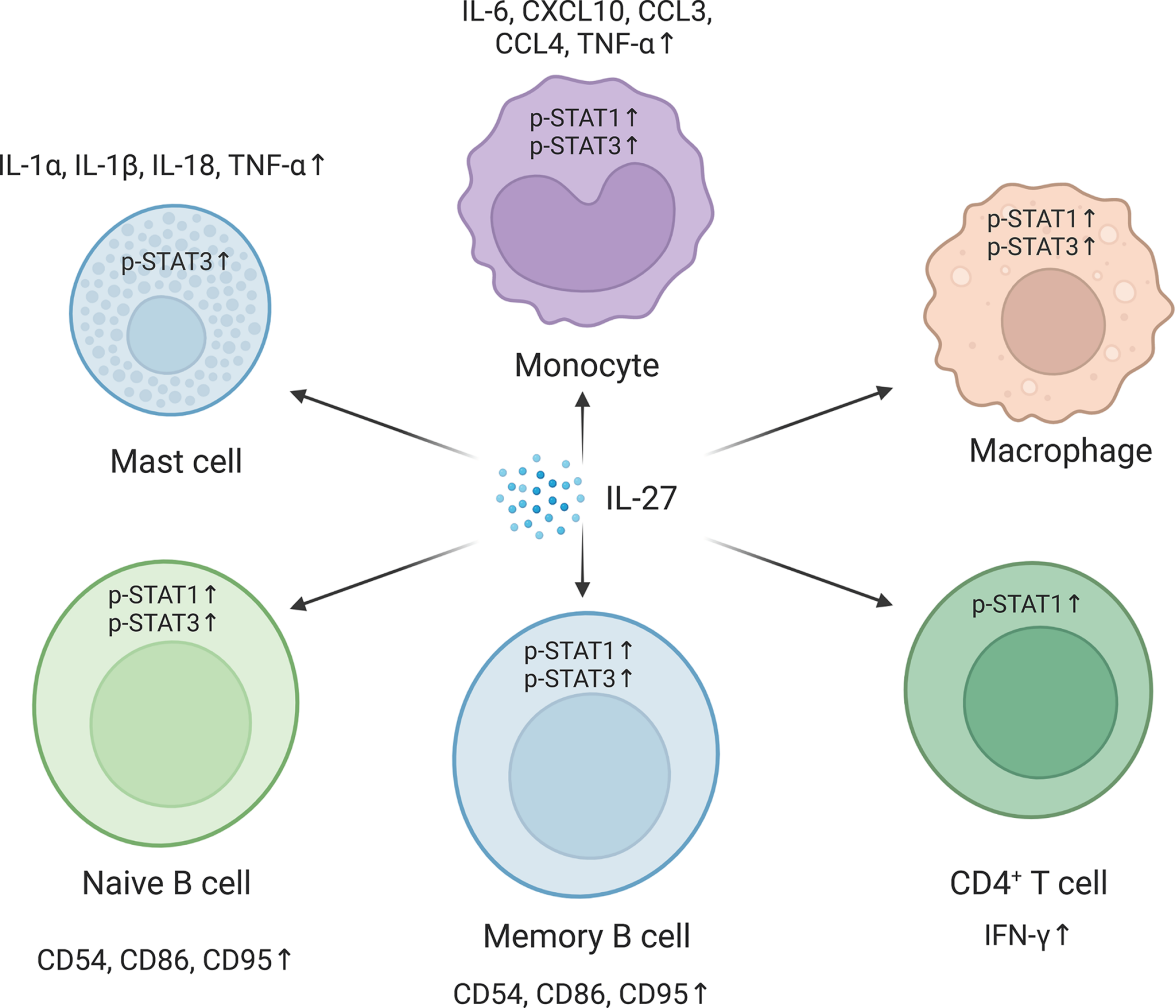
Pathways in different cells are activated in respond to the interleukin-27 (IL-27) signaling. IL-27 signaling activates signal transducer and activator of transcription 1 (STAT1) and/or STAT3 and regulates downstream gene expression. In mast cells, IL-27 signaling upregulates IL-1α, IL-1β, IL-18, and tumor necrosis factor-α (TNF-α) by activating STAT3. IL-27 promotes the phosphorylation of STAT1 and STAT3 in monocytes and macrophages and induces the expression of IL-6, C–X–C motif chemokine 10 (CXCL10), C–C motif chemokine ligand 3 (CCL3), CCL4, and TNF-α in monocytes. In naive and memory B cells, IL-27 promotes the phosphorylation of STAT1 and STAT3 and the expression of CD54, CD86, and CD95. IL-27 also improves CD4^+^ T helper type 1 (Th1) polarization of CD4^+^ T cells by increasing STAT1 phosphorylation. The figure was created with BioRender.com.

As a soluble cytokine, IL-27 primarily acts as a first messenger and mediates IL-27-induced signal transduction by binding to cell membrane-bound IL-27R. IL-27R typically activates the JAK family, and the JAK family-induced signaling pathway is considered the classical apex of the IL-27 signaling cascade  (34). The cytoplasmic region of IL-27Rα has a Box1 motif and an SH domain, which is related to JAK1 and JAK2 and may assist in signal transduction  (35). Gp130 is also associated with the JAK family members JAK1, JAK2, and tyrosine kinase (TYK) 2. The IL-27 signaling pathway can be activated by IL-27 and its receptor IL-27R in different cell types and in different cellular states (**Figure 2**). In mast cells, IL-27 treatment leads to STAT3 phosphorylation and increases mRNA levels of IL-1α, IL-1β, IL-18, and TNF-α  (28). In primary monocytes, IL-27 stimulation induces the expression of IL-6, C–X–C motif chemokine (CXCL) 10, C–C motif chemokine ligand (CCL)3, CCL4, and TNF-α, accompanied by phosphorylation of STAT1 and STAT3  (28, 36). IL-27 also increases the phosphorylation of STAT1 and STAT3 in macrophages  (37). In germinal center B cells, IL-27 induces weak phosphorylation of STAT1 and STAT3. However, IL-27 significantly promotes the phosphorylation of STAT1 and STAT3 and increases the expression of the transcription factor T-bet in naive and memory B cells. In anti-Ig-stimulated-naive or memory B cells, IL-27 also induces CD54, CD86, and CD95 expression. Moreover, IL-27 increases the proliferation of anti-Ig-activated naive B cells and anti-CD40-activated naive and germinal center B cells but not of CD40-activated memory B cells. In CD4^+^ T cells, IL-27 induces expression of the transcription factor T-bet in a STAT1-dependent and STAT1-independent manner, which is crucial for Th1 polarization. IL-27 activates different JAK family members and downstream signaling pathways in different cell types and cell states, which regulates the signaling and biological pattern of immune cells. However, the exact roles and mechanisms of IL-27-induced JAK signal transduction in different cells remain unclear, and further studies are needed.

## IL-27 Is Related to the Progression of RA

### Elevated IL-27 Levels Are Detected in RA Patients

Many studies have demonstrated that there are higher levels of IL-27 in RA peripheral circulation. Wong et al. found that RA patients exhibited higher plasma concentrations of IL-27 than healthy controls (10). Similarly, two studies by Shen et al. and Lai et al. found that IL-27 in RA serum is significantly increased, and levels of serum IL-27 are positively correlated with disease activity score 28 (DAS-28), indicating that high disease activity of RA is accompanied by high IL-27 levels  (38, 39). Moreover, Shen et al. suggested that RA-ILD patients also displayed high levels of serum IL-27 (38). After leflunomide treatment, RA patients also exhibited decreased serum IL-27 levels (39). However, Tanida et al. suggested that plasma IL-27 levels in RA and osteoarthritis (OA) patients were comparable  (11). In summary, the above studies indicated that the observed increased IL-27 levels reflect a certain degree of inflammatory status in RA patients.

Rheumatoid nodules are a common extra-articular manifestation of RA. Rheumatoid nodules are frequently present in subcutaneous pressure points and occasionally in visceral organs, such as the lung and heart (40–42). The pathological mechanism of rheumatoid nodules has been identified as granulomas driven by Th1 cells (3). Recently, Millier et al. determined that IL-27 levels in RA rheumatoid nodules are higher than that in RA synovial membranes and even higher than that in synovial membranes from end-stage RA (12). This means that rheumatoid arthritis might be a crucial source of IL-27 in RA. It is known that the occurrence of rheumatoid nodules is related to RA severity. Early RA patients who have subcutaneous rheumatoid nodules exhibit more rapid joint destruction and higher rates of hospital admission (43, 44). However, the level of IL-27 in rheumatoid nodules was studied only by one research group, and there is no greater independent evidence to document this. Therefore, it should be treated with caution. Further studies should keep exploring the level of IL-27 in rheumatoid nodules and whether IL-27 from rheumatoid nodules contributes to sustained systemic and synovial inflammation.

Studies comparing IL-27 levels in the joint cavity between RA and OA are lacking. However, increased IL-27 levels have been detected in both RA synovial membranes and synovial fluids compared to OA tissues (11, 12). Moreover, mRNA expression of the IL-27 receptor is also increased in RA synovial membranes. Increased IL-27 levels indicate that there are correlations between IL-27 and RA, meaning that the IL-27 signaling pathway might contribute to synovitis in RA. However, the possibility cannot be ruled out that IL-27 might be an innocent bystander in RA (12).

### Association Between IL-27 Gene Polymorphisms and RA

It is well known that the occurrence of complex diseases is usually affected by genetic environmental factors. An SNP is a widespread and crucial form of genetic variation that has been used to explore disease susceptibility with respect to genetic factors (45). In RA, more than 100 SNPs are related to RA susceptibility factors according to genome-wide association study (GWAS) analysis (46). An association of the IL-27 SNP with genetic susceptibility to RA in Chinese Han and Polish populations has been described, which implies that IL-27 may be associated with the occurrence and development of RA (9, 47).

### IL-27 Treatment Exacerbates or Alleviates Arthritis in Animal Experiments

Although it is unknown how exogenous IL-27 affects the progression of RA patients, validation from an arthritis animal model could help to clarify this. IL-27 plays paradoxical roles in arthritis animal models. Niedbala et al. first explored the role of IL-27 in arthritis mice and found that arthritis in IL-27-treated collagen-induced arthritis (CIA) mice was relieved (48). The intra-articular overexpression of IL-27 also attenuates arthritis severity in CIA mice (49). However, IL-27 treatment worsens arthritis in proteoglycan-induced arthritis (PGIA) mice (50). These seemingly contradictory results are reasonable when considering different pathological mechanisms in different arthritis models. Specifically, arthritis in CIA mice is primarily dominated by Th17 cell expansion but Th1 cells in PGIA mice (51, 52). Due to the distinct pathogenesis and pleiotropy of IL-27, the responses of different arthritis animal models are diametrically opposed. Evidences from arthritis animal models suggests that rather than a bystander, IL-27 may also be involved in the progression of RA.

## Cross Talk Between IL-27 Signaling and RA Pathologies

Given the available studies, we can preliminarily determine that IL-27 signaling could influence RA development by regulating CD4^+^ T cell differentiation, inhibiting monocytes/macrophages and osteoclasts in the joint cavity, interrupting synovial ectopic lymphoid structure (ELS) interactions with Th17 cells, and regulating RA synovial fibroblast (RA-FLS)-mediated inflammation. However, some conclusions from different studies are conflicting, and clinical samples or arthritis models were not utilized in some studies. To clearly present and discuss the mechanisms of IL-27 signaling and RA pathologies, a table is provided (**Table 1**).

**Table 1:** Effects and functions of IL-27 in RA different tissues and cells.

Targets of IL-27	Sources of evidence	Effects	Mechanisms	References
CD4^+^ T cell	Animals and cells	Uncertain	IL-27 promotes Th1 development and inhibits Th17 cell differentiation. However, the role of IL-27 in RA Treg development remains unclear according to the current research.	(48, 53–63)
Monocytes and macrophages in joint cavity	Clinical samples	Protective	CD14^+^ MNCs may be the main source of IL-27 in joint cavity. IL-27 might exert Th17 inhibitory function in joint cavity.	(11, 64)
Osteoclasts	Clinical samples and animals	Protective	IL-27 inhibits osteoclast differentiation by inhibiting RANK/RANKL signaling pathway.	(65, 66)
RA-FLS	Clinical samples	Uncertain	IL-27 promotes or inhibits the production of inflammatory cytokines of RA-FLS.	(10, 11)
ELS	Clinical samples and animals	Protective	IL-27 inhibits the formation of ELS by inhibiting Pdp^+^ Th17 cells.	(67, 68)

Th1, CD4^+^ T helper type 1; Th17, CD4^+^ T helper type 17; MNC, mononuclear cell; RANK, receptor activator of nuclear factor kappa-B (RANK); RANKL, RANK legend; RA-FLS, rheumatoid arthritis synovial fibroblast; ELS, ectopic-like structure.

### IL-27 Regulates CD4^+^ T Cell Differentiation

CD4^+^ T cells play a pivotal role in RA physiopathology (69). Th1 cell infiltration is traditionally believed to be crucial for RA pathogenesis due to inadequate knowledge of the CD4^+^ T cell subpopulation (69). With advances in CD4^+^ T cell research, Th17 cells have been identified as a separate CD4^+^ cell subpopulation that is closely related to autoimmune diseases (70, 71). RA has also been interpreted as a Th17-driven autoimmune disease (71). There was a positive correlation between the DAS-28 score and Th17 cells but not the Th1 cell proportion in RA peripheral blood  (71). Moreover, when discussing CD4^+^ T cells in RA, Tregs also deserve our attention. Tregs exert pivotal roles in the maintenance of immunosuppressive activity (72). However, impaired Treg function in RA patients has been observed and is considered a key pathological change (73).

**Figure 3:**
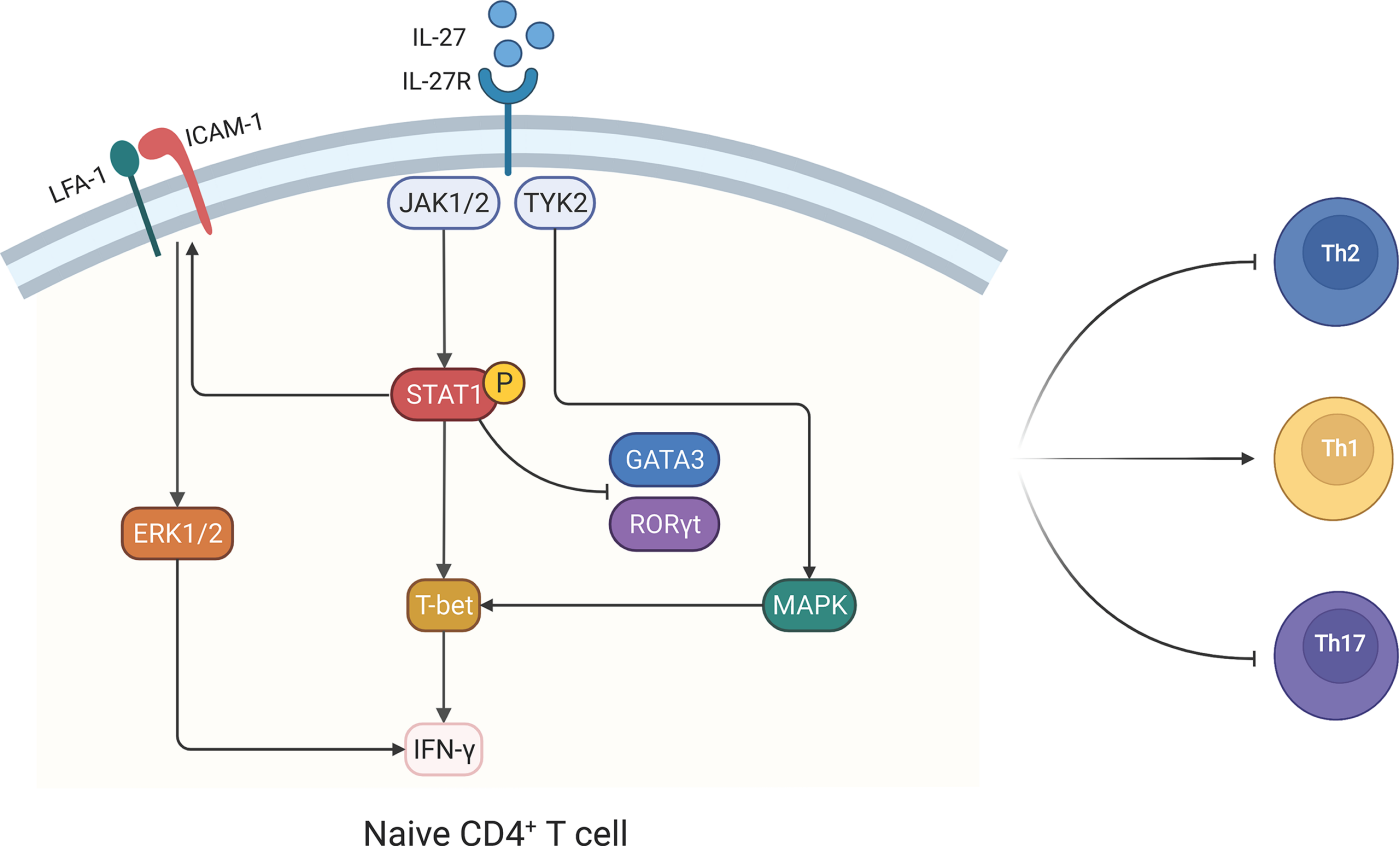
Interleukin-27 (IL-27) promotes CD4^+^ T helper type 1 (Th1) cell differentiation and inhibits Th2 and Th17 cell development in CD4^+^ T cells. IL-27 promotes Th1 development *via* multiple signaling pathways. In CD4^+^ T cells, IL-27 binds to IL-27 receptor and activates Janus tyrosine kinase 1 (JAK1), JAK2, and tyrosine kinase 2 (TYK2). Subsequently, phosphorylated signal transducer and activator of transcription 1 (STAT1) activates T-bet, and this drives naive CD4^+^ T cell to skew toward Th1 cells. STAT1 phosphorylation also promotes Th1 development *via* leukocyte function-associated antigen 1 (LFA-1)/intercellular adhesion molecule 1 (ICAM-1) and extracellular signal-regulated kinase (ERK)1/2 signaling pathway. In addition, Th1 differentiation can be improved by the TYK2/MAPK/T-bet signaling pathway, which is STAT1 independent. STAT1 phosphorylation induced by IL-27 inhibits GATA-binding protein 3 (GATA-3) and orphan receptor gamma t (RORγt) *via* a STAT1-dependent manner, which blocks naive CD4^+^ T cell differentiate into Th2 and Th17. The figure was created with BioRender.com.

The regulatory roles of IL-27 on the CD4^+^ T cell subpopulation can be summarized as follows: IL-27 promotes Th1 cells but restrains Th2 and Th17 cells (**Figure 3**). IL-27 induces Th1 cells by upregulating T-box expressed in T cell (T-bet) expression *via* STAT1 activation and activating TYK2/mitogen-activated protein kinase (MAPK)/T-bet and leukocyte function-associated antigen (LFA)-1/intercellular adhesion molecule (ICAM)-1/extracellular signal-regulated kinase (ERK)1/2 signaling pathways (53, 54). Moreover, IL-27 inhibits the expression of GATA-binding protein 3 (GATA-3) in naive CD4^+^ T cells *via* STAT1 activation, which ultimately inhibits Th2 differentiation (55). For Th17 cells, IL-27 downregulates IL-17 expression *via* retinoic acid-related orphan receptor gamma t (RORγt) inhibition by activating the STAT1 signaling pathway (56).

The role of IL-27 in Tregs exhibits double-sided and confusing effects. On the one hand, several studies have shown that the IL-27 signaling pathway promotes proinflammatory functions *via* Treg inhibition. IL-27 inhibits Treg cell generation and the expression of CD25 and cytotoxic T lymphocyte-associated antigen (CTLA)-4 under *in vitro* conditions  (57). Another *in vitro* experiment demonstrated that IL-27 suppresses the development of induced Tregs in a STAT1-independent manner  (58). In addition, blocking the IL-27 signaling pathway also alleviates mouse colitis by limiting naive CD4^+^ T cell to Treg conversion (59). On the other hand, IL-27 exerts anti-inflammatory effects in Tregs in several disease conditions. Nguyen et al. reported that Tregs inhibit effector T cell proliferation in experimental allergic airway inflammation in a lymphocyte activation gene (LAG)3-dependent manner when the IL-27 signaling pathway is activated, although Treg proliferation is also suppressed (60). Do et al. demonstrated that Treg-specific IL-27Rα deletion did not alter Treg development under *in vivo* conditions (61). Further in-depth research from Do et al. found that IL-27 signaling activation is essential for the suppressive function and stability of Foxp3^+^ Tregs in an encephalomyelitis (EAE) mouse model, which was accompanied by LAG3 upregulation. Similar to the above studies, the IL-27/LAG3 axis was found to enhance Foxp3^+^ Treg-suppressive function during intestinal inflammation (62). However, a study from Batten et al. indicated that Tregs from Il27ra deletion mice also inhibited effector T cell proliferation, indicating that the IL-27 signaling pathway is not essential for Treg-suppressive functions under physiological conditions  (63). In an arthritis model, IL-27 treatment affected the expression of IL-17 but not Foxp3 in CIA mouse spleens  (48). Regrettably, at present, no experiment has determined whether and how IL-27 signaling affects Tregs in the synovitis microenvironment in RA patients. In summary, the IL-27 signaling pathway has dual effects on Tregs. It seems that IL-27 signaling is required for Treg-suppressive functions under disease conditions *in vivo*. However, direct evidence for this is needed in RA patients and model mice.

### IL-27 Inhibits Inflammation and Differentiation of Monocytes/Macrophages in RA Joints

It seems likely that IL-27 protects RA patients from joint inflammation and joint destruction *via* the monocyte/macrophage lineage according to *in vitro* experiments. Tanida et al. found that IL-27 in the joint cavity appears to be produced by CD14^+^ mononuclear cells (MNCs) as determined by analyzing RA synovial fluid because IL27^+^ cells are primarily localized in CD14^+^ MNCs  (11). In addition, there were more IL-27^+^ MNCs in RA synovial membranes than in OA synovial membranes. Considering that CD14 is predominantly expressed in monocytes, macrophages, and DCs, we speculate that the monocyte/macrophage lineage in the joint cavity constitutes the main source of IL-27 in synovial fluids  (64). A study from Tanida et al. also demonstrated that levels of IL-27 in the joint cavity are negatively correlated with IL-17 levels, indicating that IL-27 inhibits inflammation mediated by Th17 cells in the joint cavity  (11).

Osteoclasts are supposed to differentiate from CD14^+^ monocytes  (74, 75). Osteoclastic bone erosion plays a dominant role in RA joint destruction. The receptor activator of nuclear factor kappa-B (RANK)/RANK ligand (RANKL) signaling pathway plays crucial roles in osteoclast differentiation. Activation of IL-27 signaling strongly depresses osteoclast differentiation by inhibiting RANK expression, RANKL-mediated MAPK, and NF-κB signaling pathways and by inhibiting RANKL-mediated nuclear factor of activated T cell (NFATC) 1 induction  (65). This inhibitory effect is correlated with IL-27 receptor levels. Compared to human-derived monocytes, mouse-derived monocytes express lower levels of WSX-1, which might be the reason that CD14^+^ monocyte differentiation into osteoclasts was moderately inhibited. In addition, IL-27 downregulates RANKL expression in CD4^+^ T cells in part through STAT3, which contributes to the inhibition of CD14^+^ monocyte differentiation  (66). In short, IL-27 negatively regulates osteoclast formation and bone resorption  (75), which is beneficial for arresting the progression of bone destruction.

### Is IL-27 a Pro- or Anti-Inflammatory Agent in RA-FLS?

It seems that the effect of IL-27 on RA-FLSs is paradoxical according to *in vitro* experiments. First, IL-27 increases the expression of adhesion molecules, including ICAM-1; vascular cell adhesion molecule (VCAM)-1; inflammatory chemokines; including CCL2, CXCL9, and CXCL10; and matrix metalloproteinase (MMP) 1  (10). Moreover, a combination of IL-27 and TNF-α or IL-1β treatment significantly upregulates adhesion molecules and chemokines. The above results indicate that IL-27 inhibits the RA-FLS-mediated inflammatory response. However, another *in vitro* study demonstrated that a combination of IL-27 and TNF-α or IL-17A treatment decreased RA-FLS expression of IL-6 and CCL20  (11). Taken together, the two studies indicate that IL-27 may exert anti- or proinflammatory roles on RA-FLSs in different environments. The regulation of RA-FLSs by IL-27 is complicated and might be similar to that of CD4^+^ T cells. The unique regulatory characteristics of IL-27 in RA-FLSs should be noted and explored in future research.

### IL-27 May Break the Vicious Cycle Between ELS and Th17 Cells

It is known that ELS represents a structure of lymphocyte clusters in non-lymphoid tissues, primarily consisting of B cells, T cells, and DCs. ELS is closely related to both cellular immunity and humoral immunity  (67). The synovial membrane pathological feature in approximately 40% of RA patients is follicular synovitis with ELS  (76). Pdp^+^ Th17 cells contribute to ELS formation  (77–79). Of note, IL-27 regulates the formation and development of ELS  (67). A previous study also found that in RA patients and adjuvant-induced arthritis (AIA) mice, IL-27 signaling inhibits Pdp^+^ Th17 cells, which is linked to ELS formation  (68). In light of this, IL-27 may break the proinflammatory positive feedback between ELS and Pdp^+^ Th17 cells. However, not all RA synovitis cases exhibit ELS. Comprehensive and stratified evaluation should be performed to determine the function of IL-27 in RA synovitis.

## IL-27 Signaling Represents an RA Therapeutic Target: Future Potential and Current Challenges

The IL-27 signaling pathway has not been clinically investigated as a potential target for disease treatments except in tumors. Recently, SRF388, a targeted IL-27 p38 antibody, has been evaluated for its safety and therapeutic potential for the treatment of advanced solid tumors in a phase 1, first-in-human, dose-escalation, and expansion study (NCT04374877). Solid tumor animal models and cellular experiments with bioinformatics research have demonstrated that blocking IL-27 signaling is beneficial for the treatment of hepatocellular carcinoma and renal cell carcinoma  (80, 81). The medium-term results of a phase I clinical trial demonstrated that SRF388 inhibited STAT3 phosphorylation in 12 recruited tumor patients with mild adverse reactions  (82). These results indicate that inhibiting IL-27 signaling *via* SRF388 might represent an alternative tumor treatment.

However, IL-27, a pleiotropic cytokine, yielded confusing results when attempting to alleviate RA patients by intervening in IL-27 signaling. It is difficult to see that the roles of IL-27 in regulating RA pathology are many and even contradictory. Evidence from animal studies suggests that IL-27 exerts pro- or anti-inflammatory roles in different arthritis animal models. Conflicting results have also been observed in numerous cellular experiments. Considering the limitations of animal and cellular studies, it is not currently possible to conclude whether addition or blocking of IL-27 would treat RA according to only one or multiple laboratory studies. The roles of IL-27 in cells or tissues related to RA pathology should continue to be explored using cellular and animal studies in the future. However, further studies on IL-27’s levels and roles in RA blood, joint cavities, and rheumatoid nodules by detecting clinical specimens from RA are also important. Alejandro et al. focused on the roles of IL-27 in RA patients with follicular synovitis and found that agents regulating IL-27, such as SRF388, might only be suitable for RA patients with specific clinical and pathologic characteristics  (68).

## Conclusions

Overall, the intricate links between RA immune regulation and inflammatory responses are being actively explored but are still not fully understood. Many cytokines exert beneficial or detrimental effects on RA patients by regulating immune and inflammatory responses. Among these cytokines, IL-27 is undoubtedly one of the most controversial cytokines for RA due to its pleiotropism. The awareness of immunity and inflammation is expanded while exploring the biological functions of IL-27. The pleiotropic regulatory functions of IL-27 serve as a reminder that inflammation involves a series of continuously changing states rather than simply a resting or constant state. IL-27 might be a cytokine that promotes an inflammatory state.

In the present article, we systematically reviewed and discussed the associations between RA and IL-27 by referencing previous studies. IL-27 levels are generally elevated in blood, synovial fluids, synovial fibroblasts, and even rheumatoid nodules. Nevertheless, it remains unclear whether higher levels of IL-27 are beneficial or harmful to RA patients. According to experimental research, IL-27 alleviates RA progression by inhibiting ELS formation and osteoclast differentiation. However, other laboratory evidence indicates that the roles of IL-27 in CD4^+^ T cells and RA-FLSs are complex and even conflicting, so it is difficult to draw a definitive conclusion regarding IL-27’s functions.

Considering that widely used arthritis animal models do not fully reflect the pathological characteristics of RA, use of clinical samples from RA should be considered in future studies. IL-27 might exert different roles in different RA patients; thus, the clinical characteristics, including demographic information, laboratory tests, and medication history, of RA patients should be recorded in detail. Overall, the existing evidence suggests that IL-27 exerts both pro- and anti-inflammatory effects in different ways. It is hard to say whether activating or inhibiting the IL-27 signaling pathway would be effective as an RA treatment. It is possible that some RA patients would benefit from IL-27 signaling pathway activation, while others may benefit from pathway inhibition, and still others might be unsuitable for treatment targeting the IL-27 signaling pathway. Therefore, careful attention should be given to IL-27 signaling-targeted therapy for RA to clearly understand how IL-27 signaling works in RA patients and whether RA patients would benefit from intervening in IL-27 signaling.

## Author Contributions

ST contributed to the conception of this review. LH wrote the manuscript. LH, ZC, and KY revised the manuscript. LH and KY designed and illustrated the figures. ZC, JY, TL, WL, XB, and PS performed the literature search and interpretation. ZC, YG, YL, YaH, YiH, KQ, YW, and ST reviewed and revised the manuscript. All authors contributed to the article and approved the submitted version.

## Funding

This study was supported by the National Natural Science Foundation of China (81874383, 82074267, 82174185), the Chinese Medicine Scientific Research Project of the Health Commission of Hubei Province (ZY2021Q024), and the program of Tongji-Rongcheng Center for Biomedicine (HUST).

## Conflict of Interest

The authors declare that the research was conducted in the absence of any commercial or financial relationships that could be construed as a potential conflict of interest.

## Publisher’s Note

All claims expressed in this article are solely those of the authors and do not necessarily represent those of their affiliated organizations, or those of the publisher, the editors and the reviewers. Any product that may be evaluated in this article, or claim that may be made by its manufacturer, is not guaranteed or endorsed by the publisher.
